# The role of NMDA glutamate receptors in lung injury caused by chronic long-term intermittent hypobaric hypoxia

**DOI:** 10.1590/1414-431X2023e12549

**Published:** 2023-03-24

**Authors:** M.O. Yaman, O.F. Sönmez, T. Ekiz-Yilmaz, D. Sönmez, E.E.G. Meydanlı, I. Guner, G. Sahin, N. Dariyerli, N. Yelmen

**Affiliations:** 1 Vocational School of Health Services, Istanbul University-Cerrahpaşa, Istanbul, Turkey; 2 Department of Physiology, Cerrahpaşa Medical Faculty, Istanbul University-Cerrahpaşa, Istanbul, Turkey; 3 Department of Histology and Embryology, Istanbul Faculty of Medicine, Istanbul University, Istanbul, Turkey; 4 Department of Medical Biochemistry, Istanbul Training and Research Hospital, University of Health Sciences, Istanbul, Turkey; 5 Department of Histology and Embryology, Cerrahpaşa Medical Faculty, Istanbul University-Cerrahpaşa, Istanbul, Turkey; 6 Department of Physiology, Medical Faculty, Tekirdağ Namık Kemal University, Tekirdağ, Turkey; 7 Department of Physiology, Medical Faculty, Istanbul Aydın University, Istanbul, Turkey

**Keywords:** Chronic long-term intermittent hypoxia, Lung injury, Glutamate, MK-801, NMDARs

## Abstract

Chronic intermittent hypoxia (CIH), a component of sleep apnea-hypopnea syndrome, is suggested to cause damage to lung tissue, and the role of glutamate is not well studied. We used a chronic long-term intermittent hypobaric hypoxia (CLTIHH) model of rats to find out if such procedure causes lung injury and the potential effect of N-methyl-D-aspartate receptors (NMDARs) by using receptor antagonist MK-801 (dizocilpine). Thirty-two rats were placed into four groups; a control and three CLTIHH groups where rats were placed into a low-pressure chamber set to 430 mmHg for 5 h/day, 5 days/week, for 5 weeks. Only one group received MK-801 (0.3 mg/kg, *ip*) daily. We evaluated tumor necrosis factor (TNF)-α, interleukin (IL)-6, IL-10, and nuclear factor (NF)-kB for the inflammatory process, superoxide dismutase (SOD), malondialdehyde (MDA), catalase (CAT), glutathione peroxidase (GPX), total antioxidant status (TAS), and total oxidant status (TOS) for oxidative stress, and caspase-9 levels. Blood plasma, bronchoalveolar fluid (BALF), and lung tissue extracts were evaluated. Both oxidant and inflammatory parameters were significantly increased in all the mediums of the CLTIHH groups except the group that received MK-801. Significant evidence was collected on MK-801 alleviating the effect of CLTIHH. Histological evaluations revealed lung damage and fibrotic changes in the CLTIHH groups. It was first shown that the CLTIHH procedure caused chronic lung injury, and that inflammation and oxidant stress were influential in the formation of lung injury. Secondly, NMDAR antagonist MK-801 effectively inhibited the development of lung injury and fibrosis.

## Introduction

Hypoxia is one of the most common stressors for the human body and the type of exposure can lead to different results. Intermittent hypoxia (IH) can be defined as exposure to successive periods of hypoxia and normoxia (1). IH occurs in many physiological and pathological conditions (2).

Of the different chronic intermittent hypoxias (CIH), the most common is sleep apnea-hypopnea syndrome. In sleep apnea syndrome (SAS), IH exposure is directly related to the severity of SAS. Increased extent and frequency of apneic periods in patients with severe SAS may cause these patients to remain hypoxic overnight (2,3). This type of IH can occur both in some patients with severe SAS (who are hypoxic during the night) and during repeated ascents to high altitudes (such as working at altitude and living at sea level) (4).

It is suggested that SAS causes harm to the respiratory system, cardiovascular system, and metabolic health (5). SAS includes cyclic hypoxemia and reoxygenation phases similar to ischemia and reperfusion (IR); however, the duration, frequency, and severity of hypoxia and reoxygenation phases may also cause different effects. There are few reports (6,7) that CIH causes lung injury, but the cause and mechanisms of lung injury are not fully known.

On the other hand, the importance of the glutamate signaling pathway has been emphasized in studies with different lung diseases in recent years. Studies have proven the existence of glutamate receptors and transporters in tissues that are not neuronal (8). Glutamate is suggested to play a role in pulmonary edema, bronchial asthma, small cell (9) and non-small cell (10) carcinoma, and especially the glutamate receptor N-methyl-D-aspartate receptors (NMDARs) have a critical role in these pathologies. Activation of NMDARs increases calcium entry into the cell and eventually leads to cell death. Calcium entry into the cell can cause cell death by various mechanisms including the formation of oxygen radicals (11). In light of this information, glutamate NMDARs, which cause cellular damage in neuronal tissues, may also play a role in the lungs in chronic long-term intermittent hypoxia (CLTIHH).

MK-801 is a central nervous system (CNS)-associated glutamate non-competitive NMDAR antagonist (12). Intriguingly, MK-801 has been suggested to act as a pro- and antioxidant in addition to its CNS-related anesthetic and anticonvulsant properties (13). On the other hand, it has been suggested that the high presence of NMDARs in the lung contributes to pulmonary edema, which might be alleviated by MK-801 (14-16). Moreover, studies point out that NMDAR inhibitors reduce acute injury of the lung (17,18).

### Aim

The effect and mechanism of SAS, which is a very common disease, and its component, CIH, on lung tissue are not fully understood. IH can have curative or detrimental effects on different organ systems, and the effect depends on the severity, extent, and frequency of exposure (19-22). Although a few studies recently reported lung injury caused by different IH procedures (23,24), the effects of CIH resembling severe SAS on lung tissue have not been studied extensively. In addition, the demonstration of NMDARs in many non-neuronal tissues and lungs (25) suggests that glutamate toxicity is not limited to the CNS and may cause damage to the lungs as well as distant tissues and organs through the activation of NMDARs with as yet unknown mechanisms.

Therefore, we aimed to examine the lung tissue in a severe SAS-like rat model (19,26). In addition, the role of the glutamatergic pathway on this possible injury was examined by using glutamate receptor antagonist MK-801.

## Material and Methods

In this study, 32 male adult Sprague-Dawley (245-312 g) rats were randomly assigned to 4 groups of 8 each. In the control group, rats were kept in the same place and conditions as all other groups but stayed in a regular cage. In the CLTIHH group, rats were placed into the low-pressure chamber to attain CLTIHH. The pressure of the chamber was stabilized at 430 mmHg (corresponding to an altitude of 4572 m above sea level) by an adjustable valve and a dry vacuum pump. The CLTIHH procedure was performed for 5 h a day (from 09:00 am to 02:00 pm), 5 days per week, and for 5 weeks in total. The temperature of the chamber was kept at 28°C with 12-h light/dark cycles. The hypobaric chamber was opened at the same hour every day for daily care of the rats. In the CLTIHH+SALINE group, the rats were placed in the chamber under the same conditions as the previous group, but they were given saline injections daily (0.5 mL, *ip*). The purpose of saline injection in this group was to rule out the possible effects of injection stress on the results. Finally, in the CLTIHH+MK-801 group, the rats were placed in the chamber under the same conditions as the 2 previous groups, but they were given MK-801 (dizocilpine) daily (0.3 mg/kg, 0.5 mL in volume/w saline, *ip*). We made certain that the saline injections followed the exact MK-801 regimen to minimize any inconsistencies. Both saline and MK-801 injections were made in a head-down position and the injection site was carefully chosen in the caudal abdominal quadrants. The daily injections alternated between the right and left sides.

After 5 weeks of CLTIHH protocol, rats were anesthetized with 35 mg/kg sodium pentobarbital in the peritoneum. Tracheostomy was immediately performed followed by catheterization with a 14-caliber blunt tip catheter to keep airways open. The same catheter was then used for bronchoalveolar lavage fluid (BALF) collection (4 mL of saline applied to lungs and withdrawn, 3 times). Blood was collected by cardiac puncture, and finally the rats were euthanized by cervical dislocation. The upper lobes of the lungs were processed to evaluate oxidative stress markers and cytokine levels, the middle lobes for histopathological examination, and the lower lobes were used to determine the wet/dry ratio. The samples for biochemical analysis were stored at -80°C, and the middle lobe samples were placed in neutral formalin.

Of each upper lobe, 150 mg were homogenized and processed (27). The instructions of the commercial Elisa kits were followed. For inflammatory evaluation, tumor necrosis factor-alpha (TNF-α), nuclear factor kappa B (NF-kB), interleukin-6 (IL-6), and interleukin-10 (IL-10) were evaluated. For oxidative stress, superoxide dismutase (SOD), total oxidant status (TOS), total antioxidant status (TAS), malondialdehyde (MDA), catalase (CAT), and glutathione peroxidase (GPX) levels were determined. As an indicator of apoptosis, caspase-9 levels (inactive) were measured.

Lower lobes were weighed wet and after drying to assess tissue edema. Tissue slides were examined and photographed under a light microscope (Olympus BX61, Japan), and two independent histologists assessed and scored the tissue injuries in a blinded fashion. Five parameters of lung injury (interstitial edema/infiltration, intra-alveolar edema/infiltration, intra-alveolar hemorrhage, capillary congestion, and airway epithelial cell damage) were evaluated, and the total lung injury score was calculated according to Yaman et al. (28). In addition, the Modified Ashcroft scoring method was used to evaluate fibrosis on Masson-stained slides (29).

The National Institutes of Health Guide for Care and Use of Laboratory Animals and the Helsinki Declaration were strictly followed in all experimental procedures, which were approved by the Animal Care and Use Ethics Committee of Istanbul University (No. 105711).

Regarding Elisa Kits, TAS and TOS kits were manufactured by SUNRED (Shanghai Sunred Biological Technology Co., Ltd., China) and all the others were manufactured by Elabscience Biotechnology Inc. (USA).

### Statistical analysis

The Kolmogorov-Smirnov test was performed to assess if data of the measured parameters met the requirements of a normal distribution. As the data distribution met the criteria for normality, the homogeneity of variances was checked using the Levene's test, and one-way ANOVA and Bonferroni correction tests were employed using the latest available version of GraphPad Prism software (USA). P<0.05 was considered significant.

## Results

Wet/dry ratios revealed the formation of edema in CLTIHH exposed animals. The levels of the CLTIHH groups were significantly elevated compared to the control group. The wet/dry ratios of the CLTIHH group treated with MK-801 were not changed significantly compared to the control (Table 1).

**Table 1:** Lung wet/dry ratio.

Parameter	Control	CLTIHH	CLTIHH+SALINE	CLTIHH+MK-801
Lung wet/dry ratio	4.48±0.96	9.03±01.11**	8.76±1.29**	6.19 241 1.32

Data are reported as mean±SE. **P<0.005 *vs* control (ANOVA ). CLTIHH: chronic long-term intermittent hypobaric hypoxia.

Antioxidant levels of the enzymes SOD, GPX, and CAT decreased in the blood, BALF, and lung tissue samples in the CLTIHH and CLTIHH+SALINE experimental groups, thus TAS levels decreased, while TOS and MDA levels increased significantly (Tables 2-4).

**Table 2:** Parameters studied in blood samples.

Parameter	Control	CLTIHH	CLTIHH+SALINE	CLTIHH+MK-801
SOD (ng/mL)	2.04±0.15	1.29±0.10**	0.91±0.15***	0.63±0.16**^#^
MDA (nmol/mL)	4.51±0.17	6.70±0.24***	7.53±0.27***	4.09±0.21^###&&&^
GPX (pg/mL)	724.09±21.71	535.75±36.38**	550.96±27.05**	743.33±26.25^##&&^
CAT (ng/mL)	26.94±1.46	13.38±1.42***	11.36±1.07***	17.32±0.57***^&&^
TAS (U/mL)	10.05±0.30	7.43±0.20***	7.53±0.16***	10.13±0.33^###&&&^
TOS (nmol/mL)	2.71±0.09	4.08±0.08***	4.03±0.19***	2.98±0.23^#$^
CASP9 (ng/mL)	1.11±0.11	1.84±0.09**	1.81±0.17*	0.78±0.12^###&&^

Data are reported as means±SE. *vs control, ^#^ *vs* CLTIHH, ^&^ *vs* CLTIHH+SALINE, *^/#/&^P<0.05, **^/##/&&^P<0.005, ***^/###/&&&^P<0.001 (ANOVA ). CLTIHH: chronic long-term intermittent hypobaric hypoxia; SOD: super oxide dismutase; MDA: malondialdehyde; GPX: glutathione peroxidase; CAT: catalase; TAS: total antioxidant status; TOS: total oxidant status; CASP9: caspase-9.

**Table 3 t03:** Parameters studied in bronchoalveolar lavage fluid.

Parameter	Control	CLTIHH	CLTIHH+SALINE	CLTIHH+MK-801
SOD (ng/mL)	0.06±0.00	0.08±0.01	0.17±0.08	0.07±0.00
MDA (nmol/mL)	6.84±0.39	9.46±0.66*	11.55±0.38***	10.36±0.39***
GPX (pg/mL)	1213.60±91.61	454.33±24.84***	573.73±038.91***	958.89±29.13^###&&&^
CAT (ng/mL)	36.95±0.86	19.81±0.64***	21.61±1.00***	33.29±1.11^###&&&^
TAS (U/mL)	9.99±0.35	8.13±0.23**	8.17±0.17**	9.82±0.25^##&&^
TOS (nmol/mL)	3.52±0.11	4.59±0.10***	4.68±0.21**	3.04±0.06*^###^
CASP9 (ng/mL)	55.33±1.89	81.47±2.28***	108.04±6.06***^#^	74.65±3.13**^&&^

Data are reported as means±SE. *vs control, ^#^ *vs* CLTIHH, ^&^ *vs* CLTIHH+SALINE, *^/#/&^P<0.05, **^/##/&&^P<0.005, ***^/###/&&&^P<0.001 (ANOVA). CLTIHH: chronic long-term intermittent hypobaric hypoxia; SOD: super oxide dismutase; MDA: malondialdehyde; GPX: glutathione peroxidase; CAT: catalase; TAS: total antioxidant status; TOS: total oxidant status; CASP9: caspase-9.

**Table 4:** Parameters studied in lung tissue samples.

Parameter	Control	CLTIHH	CLTIHH+SALINE	CLTIHH+MK-801
SOD (ng/mL)	21.75±2.04	11.16±0.78**	12.69±1.22*	20.66±1.62^##&^
MDA (nmol/mL)	17.14±0.63	22.79±0.69***	25.04±1.19***	14.42±1.50^##&&&^
GPX (pg/mL)	18.50±0.86	11.38±0.50***	12.83±1.04**	18.64±1.27^##&^
CAT (ng/mL)	75.10±1.26	50.47±1.47***	48.65±1.12***	66.30±1.76*^###^
NFkB (pg/mL)	4.77±0.11	6.98±0.19***	7.33±0.11***	5.03±0.11^###&&&^
TNF-α (pg/mL)	0.23±0.02	0.47±0.01***	0.48±0.02***	0.21±0.01^###&&&^
IL-6 (pg/mL)	2.07±0.09	3.36±0.16***	3.19±0.10***	2.42±0.13^##&&^
IL-10 (pg/mL)	0.93±0.07	0.44±0.02***	0.31±0.02***^#^	0.96±0.06^###&&&^
TAS (U/mL)	3.34±0.10	1.87±0.09***	2.07±0.04***	2.90±0.11^###&&&^
TOS (nmol/mL)	6.19±0.19	7.92±0.20***	8.27±0.13***	6.99±0.18^#&&&^
CASP9 (ng/mL)	2.39±0.12	3.60±0.14***	3.43±0.18**	2.49±0.17^##&^

Data are reported as means±SE. *vs control, ^#^ *vs* CLTIHH, ^&^ *vs* CLTIHH+SALINE, *^/#/&^P<0.05, **^/##/&&^P<0.005, ***^/###/&&&^P<0.001 (ANOVA). CLTIHH: chronic long-term intermittent hypobaric hypoxia; SOD: super oxide dismutase; MDA: malondialdehyde; GPX: glutathione peroxidase; CAT: catalase; NFkB: nuclear factor kB; TNF-α: tumor necrosis factor alpha; IL: interleukin; TAS: total antioxidant status; TOS: total oxidant status; CASP9: caspase-9.

In terms of oxidant/antioxidant balance, the general trend was towards a suppressing effect of oxidative stress. The SOD and CAT levels of the MK-801-treated group did not reach the control group levels and remained significantly lower, while the TAS and TOS ratios were not significantly different from the control group in the blood samples. The TOS ratio in the plasma was significantly higher than the control group. MDA levels in BALF samples were higher than the control group. When the results of the lung tissue samples were examined, only the CAT levels showed a significant difference compared to the control group (Table 2-4). Both TAS and TOS levels did not differ significantly compared to the control group. In addition, caspase-9 levels in this group did not differ significantly from the control group, except in the BALF measurements. IL-10 levels of the CLTIHH and CLTIHH+SALINE experimental groups were significantly decreased. No significant difference was found between the levels of TNF-α, IL-6, and NFkB in the CLTIHH+MK-801 and control groups. The results of the CLTIHH and CLTIHH+SALINE were significantly different than those of the control group (Table 2-4).

**Figure 1:**
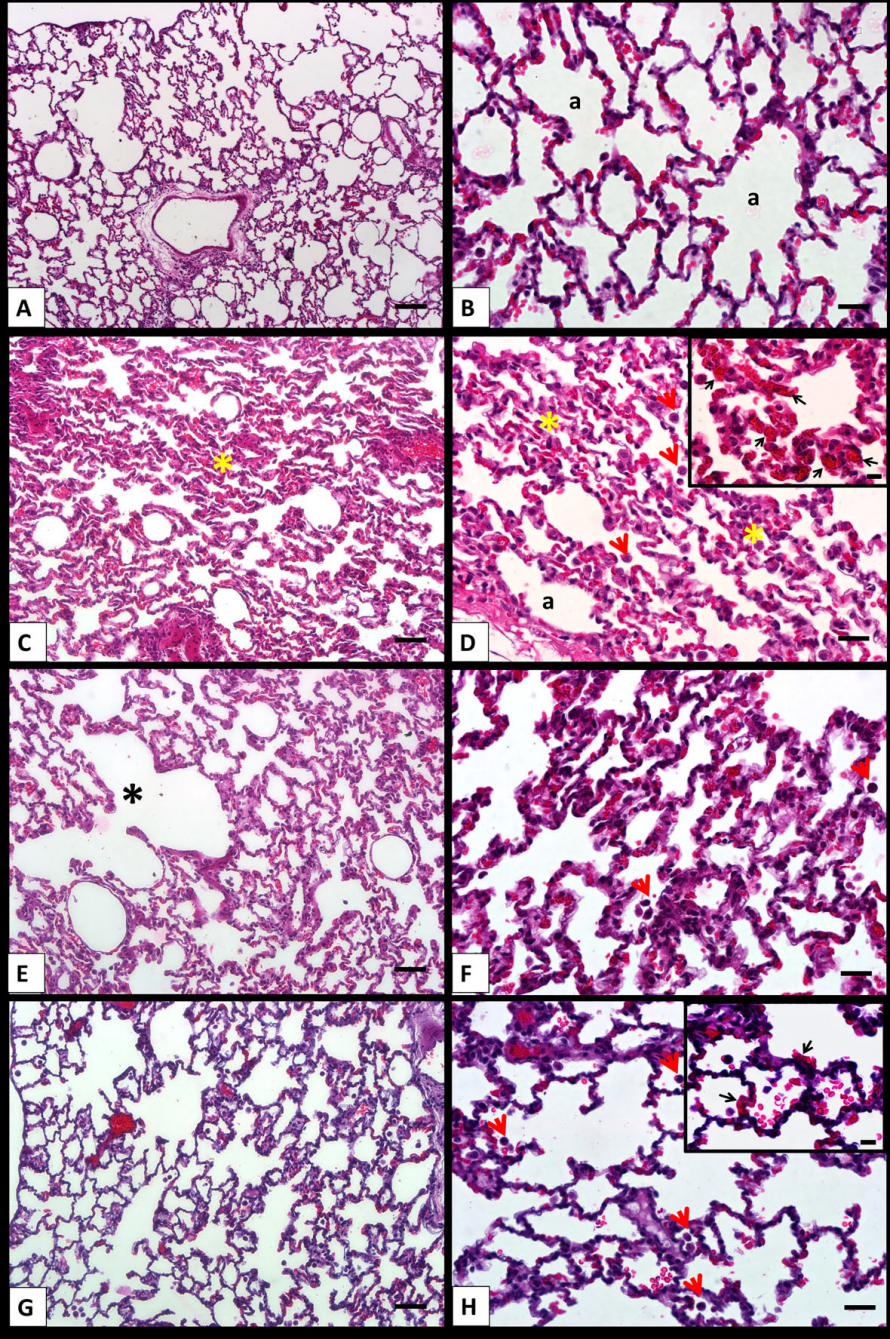
Histological sections of lung samples from control (**A** and **B**), chronic long-term intermittent hypobaric hypoxia (CLTIHH) (**C** and **D**), CLTIHH+SALINE (**E** and **F**), and CLTIHH+MK-801(**G** and **H**) groups stained with H&E. Histological changes of the control group samples were minimal. Alveolar walls are thin, and most alveoli contain few cells (a, alveoli). Histological features such as collapsed alveoli (yellow asterisks), intra-alveolar macrophages (short tailed red arrows) (**D**), capillary congestion (**D**, inset; black arrows), and emphysema-like areas (black asterisk) are seen in CLTIHH and CLTIHH+SALINE groups. The CLTIHH+MK-801 group also has intra-alveolar macrophages (short tailed red arrows), capillary congestion (**H**, inset; black arrows), and intra-alveolar hemorrhage. Scale bars for the left column are 100 µm and 50 µm for the right column. Scale bars for the insets are 50 µm.

**Figure 2:**
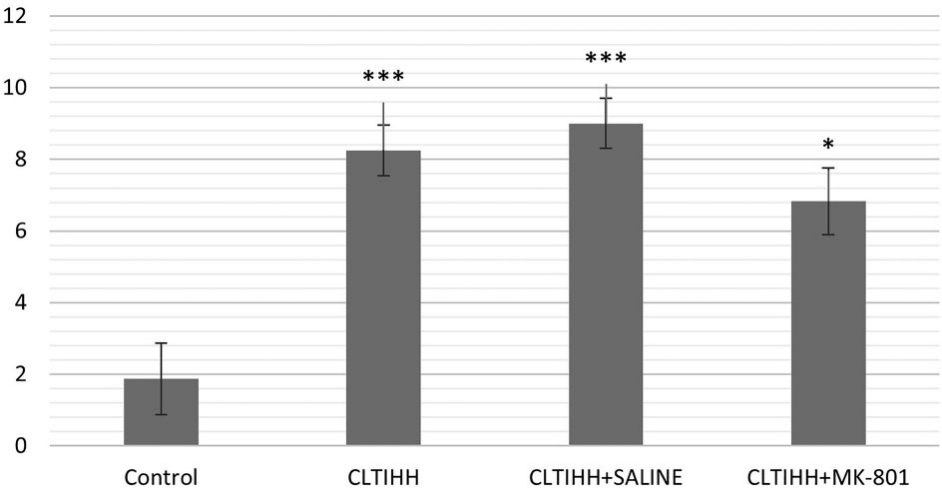
Histological score of the study groups. Lung injury in rats was induced by chronic long-term intermittent hypobaric hypoxia (CLTIHH) and then rats received saline or MK-801. *P<0.05, ***P<0.001 *vs* control (ANOVA).

Histological evaluations of the lung tissue from the CLTIHH and CLTIHH+SALINE experimental groups indicated moderate lung damage. Thickening of the alveolar walls in some areas, inflammatory cell infiltration, and formation of interstitial edema with capillary congestion were prominent changes. In addition, when compared to the control group, intra-alveolar hemorrhage was significantly increased in the CLTIHH+SALINE experimental group (Figure 1). In the CLTIHH+MK-801 group, capillary congestion, interstitial edema, inflammatory infiltration, and intra-alveolar edema scores were lower, but not significantly different from the other CLTIHH groups (Figure 2).

**Figure 3:**
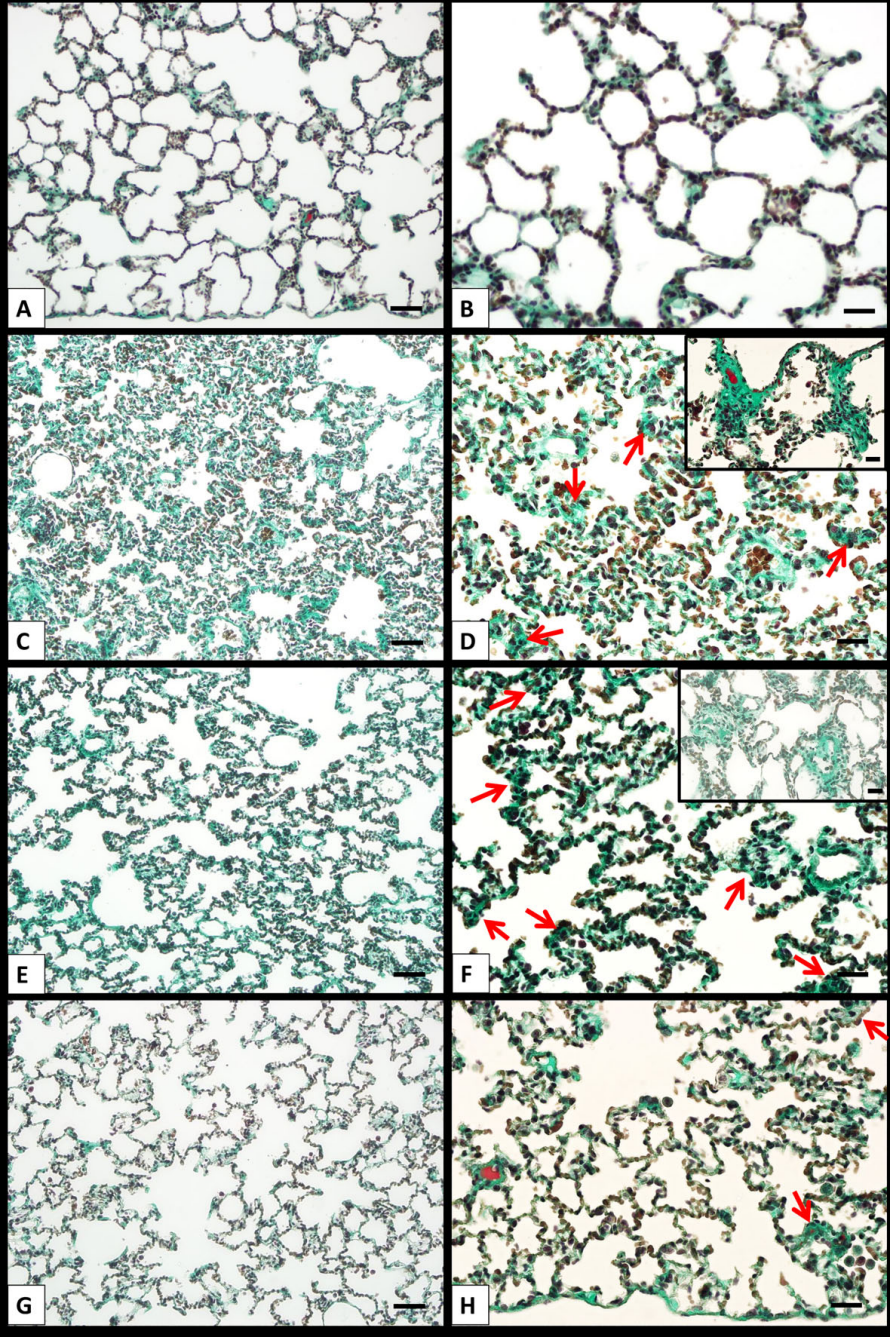
Lung injury in rats was induced by chronic long-term intermittent hypobaric hypoxia (CLTIHH) and then rats received saline or MK-801. Lung samples of the control (**A** and **B**), CLTIHH (**C** and **D**), CLTIHH+SALINE (**E** and **F**), and CLTIHH+MK-801 (**G** and **H**) groups were stained with Masson's trichrome. In the control group, thin small fibers are seen in some alveolar walls that do not contain fibrotic areas. Adjacent fibrotic walls at the alveolar septa (long tailed red arrows) and single fibrotic masses in the CLTIHH and CLTIHH+SALINE groups are observed in most of the area (inset). Scattered fibrotic changes in the alveolar walls were not connected in the CLTIHH+MK-801 group. Scale bars for the left column are 100 µm and 50 µm for the right column. Inset: Scale bars for the insets are 50 µm.

**Figure 4:**
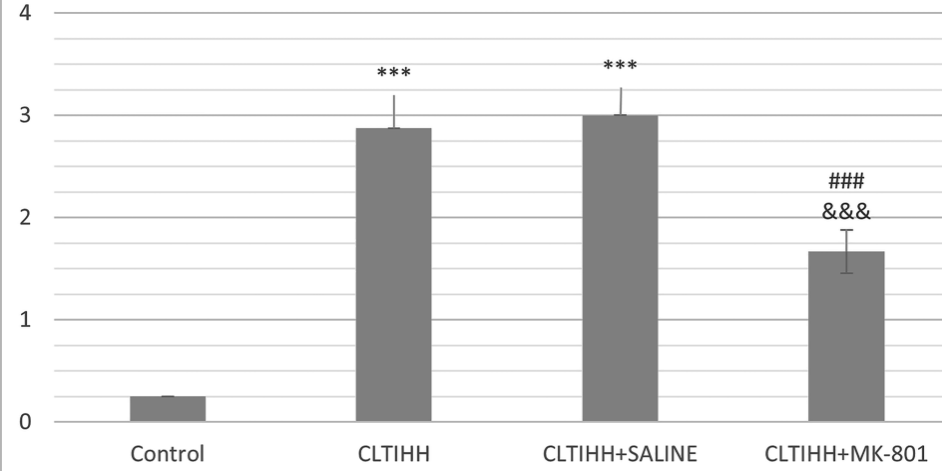
Fibrosis score of the study groups. Lung injury in rats was induced by chronic long-term intermittent hypobaric hypoxia (CLTIHH), and then rats received saline or MK-801. ***P<0.001 *vs* control, ^###^P<0.001 *vs* CLTIHH, ^&&&^P<0.001 *vs* CLTIHH+SALINE (ANOVA).

Significant fibrotic changes were evident in the CLTIHH groups. Adjacent fibrotic walls were observed in the alveolar septa in most of the areas in the CLTIHH and CLTIHH+SALINE experimental groups. Single fibrotic masses were rarely observed in some sections (Figure 3). Scattered fibrotic changes in the alveolar walls were not connected with each other in the CLTIHH+MK-801 group. Scoring of fibrosis clearly demonstrates that the CLTIHH procedure induced fibrotic changes and MK-801 administration alleviated the changes (Figure 4).

## Discussion

The effects of IH vary according to the frequency, depth, and duration of hypoxic exposure. In this study, using the severe sleep apnea syndrome (SAS)-like CLTIHH (26,30) model, we aimed to investigate the effect on rat lung tissue and the role of glutamatergic pathways in the formation mechanism of this effect. Acute lung injury and pulmonary fibrosis developed in rats in both the CLTIHH and CLTIHH+SALINE groups. There are studies suggesting that CIH increases epithelial and endothelial cell damage (31) and causes inflammation and fibrosis in the lung tissue (1,5). Wang et al. (32) showed damage to rat lungs by applying cyclic CIH and suggested that both inflammatory and oxidative processes are responsible for this damage. Although we applied a different CIH model in our study, we obtained similar results. On the other hand, using a model closer to our model, Gao et al. (33) reported that CIHH can downregulate the MCT-stimulated expression of TNFα and IL-6 in the pulmonary arterial hypertension (PAH) rat model and they obtained almost opposite results to our findings. They found that their CIHH procedure reduced the intensity of the inflammatory process and proposed a therapeutic function via suppression of the NF-kB and P38 MAPK pathways. While they applied a hypoxic procedure after creating PAH, we used perfectly healthy rats to assess the effect of CLTIHH. Another possible explanation for this contradiction may be the difference between the CIH models. According to their experimental design, the hypoxia chamber was flushed with room air and 100% N_2_ to maintain 10% O_2_. In contrast, we removed the air from the chamber to reach a more realistic hypobaric environment.

Our results are further strengthened by histological evaluation, which is consistent with the increase in levels of inflammatory cytokines, oxidative, and apoptotic parameters identified in our study. Histological evaluations of the CLTIHH and CLTIHH+SALINE groups indicated moderate lung damage. Thickening of the alveolar walls in some areas, inflammatory cell infiltration, and formation of interstitial edema with capillary congestion were observed. In addition, when compared to the control group, intra-alveolar hemorrhage was significantly increased in the CLTIHH+SALINE group.

It is known that excessive accumulation of proinflammatory cytokines may cause pulmonary edema and alveolar hemorrhages (17). This is an indication of damage to the lung tissue. The development of the inflammatory process after tissue damage is an expected situation. According to the results of our study, lung tissue NFkB, TNF-α, IL-6 levels, which are inflammatory markers, increased significantly in the CLTIHH and CLTIHH+SALINE groups compared to the control group.

The inflammatory process determines how the healing/repair process will develop. Depending on the cause of the damage, its size, and the development of the inflammatory process, it may lead to a normal healing/repair response or it may be dragged towards a pro-fibrotic process. The pro-fibrotic process causes fibroblast proliferation and collagen deposition (34). IL-10 (35), which is considered an anti-inflammatory cytokine, is secreted by T-reg cells and suppresses local chemokine production. IL-10 is accepted as an important mediator in the normal healing/repair process. According to our results, IL-10 levels of the CLTIHH and CLTIHH+SALINE experimental groups were significantly decreased. This situation explains the significant fibrotic changes that were seen in the histological evaluation. Adjacent fibrotic walls were observed in the alveolar septa in most of the areas in the CLTIHH and CLTIHH+SALINE groups. Single fibrotic masses were rarely recorded in some sections. In addition, significantly higher caspase-9 levels measured in these groups indicated increased apoptotic activity during the healing/repair process.

Our lungs are the organs that are most affected by oxidative stress. General findings in the literature reveal that oxidative stress is triggered, and the production of reactive oxygen species (ROS) is increased in IH models (31,32). Oxidant/antioxidant balance changes trigger different redox-sensitive transcription factors, especially NF-kB, and affect the genetic regulation of pro-inflammatory mediators and antioxidant mechanisms (36). In their study with the cyclic CIH model, Lu et al. (31) found a significant decrease in SOD and CAT levels, which are described as antioxidant defense enzymes, and a significant increase in MDA levels as a lipid peroxidation product. Again, in a study using the cyclic IH model, Wang et al. (37) showed the change in oxidative stress level at different hypoxia depths and revealed that oxidative stress markers increased as the depth of hypoxia increased. In our study, antioxidant enzyme levels (SOD, GPX, and CAT) decreased in the blood, BALF, and lung tissue samples in the CLTIHH and CLTIHH+SALINE experimental groups, thus TAS levels decreased, while TOS and MDA levels increased significantly. Therefore, we can say that the CLTIHH model we applied caused lung damage by triggering both inflammatory and oxidative processes.

Glutamate, which is commonly found in our CNS as an excitatory neurotransmitter, performs its physiological effects not only on the nervous system but also by binding to its receptors in many organs in the body. Glutamate receptors are divided into ionotropic (iGlu) and metabotropic (mGlu) receptors. Ionotropic receptors enable glutamate to exert its physiological effect by controlling Ca^+2^, Na^+^, and K^+^ currents (8). Dickman et al. (25) suggested that the induction of NMDARs in the lungs, which belong to the iGlu receptor family, causes excitotoxicity and is responsible for the formation of pulmonary edema. They also suggested that endogenous NMDAR activation causes lung tissue damage by increasing oxidative stress. Da Cunha et al. (14) investigated the efficacy of NMDARs by designing an experimental sepsis-mediated (cecal ligation and perforation) lung injury model and administering MK-801 (dizocilpine), an NMDAR antagonist, at 4 and 7 h following perforation. According to their findings, the application after 4 hours reduced lung damage, but the application after 7 h was ineffective. In addition, Hamasato et al. (38) suggested that the use of MK-801 created curative effects in their study on the allergic lung inflammation model.

We investigated what role the NMDAR pathway might play in lung injury induced by CLTIHH. Histological examination showed that although the capillary congestion, interstitial edema, inflammatory infiltration, and intra-alveolar edema scores were low in the CLTIHH+MK-801 group, a significant difference was not found between the other CLTIHH groups. When the CLTIHH+MK-801 group was compared with the control group, no significant difference was found between the levels of TNF-α, IL-6, and NFkB. Similarly, in the CLTIHH and CLTIHH+SALINE groups, which did not receive MK-801, these markers were not significantly different from the control group. Therefore, it can be argued that MK-801 given just before CLTIHH exposure suppresses the inflammatory process. The level of IL-10 approached the levels of the control group, and it is probable that the regulatory effect of IL-10 played a part in this process.

### Conclusions

In conclusion, we showed that a 5-week long CLTIHH exposure led to lung tissue damage and fibrosis development due to oxidative stress and inflammatory processes involving the NMDAR pathway.

We also found that NMDAR antagonist MK-801 inhibited lung fibrosis and reduced other indicators of lung injury. In light of these findings, we suggest that not only glutamate NMDARs were effective in oxidative stress and inflammation due to the NF-kB pathway in lung injury caused by CLTIHH, but the series of events triggered by glutamatergic NMDARs were effective in the development of fibrosis, especially in lung tissue.

Therefore, therapeutic strategies could be developed with NMDAR antagonists for the treatment of pulmonary fibrosis and lung damage that may develop in severe SAS and in various processes that may cause the development of fibrosis in the lungs.
